# The attenuation of neurological injury from the use of simvastatin after spinal cord ischemia-reperfusion injury in rats

**DOI:** 10.1186/s12871-018-0496-6

**Published:** 2018-03-27

**Authors:** Jung-Hee Ryu, Jin-woo Park, Jin-Young Hwang, Seong-Joo Park, Jin-Hee Kim, Hye-Min Sohn, Sung Hee Han

**Affiliations:** 1 Department of Anesthesiology and Pain Medicine, Seoul National University College of Medicine, Seoul, South Korea; 2 Department of Anesthesiology and Pain Medicine, Seoul National University Bundang Hospital, Seong-nam, South Korea; 3 Department of Anesthesiology and Pain Medicine, SNU-SMG hospital, Seoul, South Korea

**Keywords:** Neuroprotection, Simvastatin, Reperfusion injury, Thoracoabdominal aortic surgery

## Abstract

**Background:**

Spinal cord ischemic injury remains a serious complication of open surgical and endovascular aortic procedures. Simvastatin has been reported to be associated with neuroprotective effect after spinal cord ischemia-reperfusion (IR) injury. The aim of this study was to determine the therapeutic efficacy of starting simvastatin after spinal cord IR injury in a rat model.

**Methods:**

In adult Sprague-Dawley rats, spinal cord ischemia was induced using a balloon-tipped catheter placed in the descending thoracic aorta. The animals were then randomly divided into 4 groups: group A (control); group B (0.5 mg/kg simvastatin); group C (1 mg/kg simvastatin); and group D (10 mg/kg simvastatin). Simvastatin was administered orally upon reperfusion for 5 days. Neurological function of the hind limbs was evaluated for 7 days after reperfusion and recorded using a motor deficit score (MDS) (0: normal, 5: complete paraplegia). The number of normal motor neurons within the anterior horns of the spinal cord was counted after final MDS evaluation. Then, the spinal cord was harvested for histopathological examination.

**Results:**

Group D showed a significantly lower MDS than the other groups at post-reperfusion day 1 and this trend was sustained throughout the study period. Additionally, a greater number of normal motor neurons was observed in group D than in other groups (group D 21.2 [3.2] vs. group A: 15.8 [4.2]; group B 15.4 [3.4]; and group C 15.5 [3.7]; *P* = 0.002).

**Conclusions:**

The results of the current study suggest that 10 mg/kg can significantly improve neurologic outcome by attenuating neurologic injury and restoring normal motor neurons after spinal cord IR injury.

## Background

Spinal cord ischemia remains to be the most impressive and devastating complication following thoracoabdominal aortic aneurysm (TAAR) surgery. Spinal cord ischemia leads to paraparesis and paraplegia, which have a major invalidating impact on the patient’s life [1]. Recently, thoracic endovascular aortic repair (TEVAR) has seen great improvement in the treatment of patients with thoracic aortic disease due to its decreased overall morbidity and mortality compared with open thoracic surgery [2]. Despite its advantages with respect to safety issues compared with conventional open surgical repair, TEVAR has been associated with spinal cord ischemia as a major complication caused by endovascular coverage or injury to the spinal cord collateral vessels [3].

The precise pathophysiology of spinal cord ischemia after TAAR surgery is unclear and probably multifactorial, including intercostal artery occlusion, spinal cord hypoperfusion during aortic occlusion, or spinal cord reperfusion [4]. Therefore, to date, a variety of surgical techniques have focused on maximizing spinal cord perfusion, oxygen delivery, and reducing oxygen consumption to minimize risk of spinal cord ischemia. These methods include systemic cooling or regional spinal cord cooling, cerebrospinal fluid (CSF) drainage, re-anastomosis (re-joining) of intercostal arteries, monitoring somatosensory-evoked potentials, and distal aortic perfusion [5]. Despite various surgical techniques, the complete elimination of spinal cord ischemia remains to be challenging, and the use of medical adjuncts for spinal cord protection may be one of the preventive or management measures of spinal cord ischemia [4]. Much progress has been made in the development of pharmacologic treatment of patients with spinal cord ischemia to minimize neurologic sequale and improve neurologic prognosis. To be clinically used, however, these neuroprotective drugs require the development of safety and pharmacokinetic profile in humans. Currently, intravenous high dose methylprednisolone remains a widespread option for acute spinal cord ischemia despite its limited efficacy and safety profile [6].

Simvastatin (Zocor®), a hydroxymethylglutaryl coenzyme-A (HMG-CoA) reductase inhibitor and lipid-lowering drug, has recently been recognized to possess powerful immunomodulatory and anti-inflammatory properties. Extensive studies with various experimental models have established the beneficial effects of simvastatin on ischemic-reperfusion (IR) injury in various organs and tissues, including intestine, heart, lungs, liver, and kidneys [7–11]. Additionally, neuroprotective effects of simvastatin have been investigated with preclinical animal models of a variety of neurologic conditions, including stroke, and traumatic brain injury [12, 13]. The preventive effect of simvastatin on neurologic damage prior to IR injury was demonstrated in a rat spinal cord ischemia model [14, 15] but its treatment effect after spinal cord IR injury has not been fully investigated. Post-treatment studies provide greater clinical implications than the pre-treatment study since trauma or injury is unpredictable. Therefore, we hypothesized that simvastatin is able to reduce neurologic injury after spinal cord IR injury. This study was designed to evaluate the treatment effect of simvastatin with various doses on neurologic damage using a rat spinal cord IR injury model.

## Methods

### Animal care and preparation

The experimental protocol of this study was approved by the Institutional Animal Care and Use Committee of Seoul National University Bundang Hospital. Animal experiments and care were conducted in compliance with the Guide for the Care and Use of Laboratory Animals, published by the US National Institutes of Health. All animals were kept at room temperature with equal lighting control (12-h light/12-h dark cycle) and all the surgical procedures and post-reperfusion neurological assessment were performed at 10 am.

### Group assignment

Forty male Sprague-Dawley rats weighing 300 to 350 g aged around 8 weeks were randomly assigned to 1 of the 4 groups before the surgical preparation. 1) A group (*n* = 10): control group with 1 ml of saline; 2) B group (*n* = 10): 0.5 mg/kg of simvastatin (Zocor®, Merck, Whitehouse Station, USA) mixed with normal saline 1 ml; 3) C group (*n* = 10): 1 mg/kg of simvastatin mixed with normal saline 1 ml; 4) D group (*n* = 10): 10 mg/kg of simvastatin mixed with normal saline 1 ml. Besides the 4 experimental groups, a blank control group without IR injury (sham group, *n* = 10) was added for each experiment. Daily oral administration was performed via oral gavage using a 16-gauge feeding needle immediate after reperfusion injury for 5 days.

### Anesthesia and surgical preparation

Anesthesia was induced in an acrylic chamber with 5 vol% isoflurane in 100% oxygen. Then, maintenance of anesthesia was done with a facial mask of inhaled 1.0–2.5 vol% isoflurane and oxygen flow of 2 L/min.

Rats were placed in the supine position and then the hair in the neck and left inguinal area were shaved. The left femoral artery was exposed for the induction of spinal cord ischemia and the tail artery was cannulated with a polyethylene catheter (PE-50) for heparin injection and monitoring of distal arterial pressure. The left carotid artery was cannulated with a 20-gauge catheter (BD Insyte, Becton Dickinson, Sandy, UT, USA) and connected to a saline-filled external blood reservoir to drain blood during the aortic occlusion period.

### Experimental protocol (spinal cord ischemia)

Spinal cord ischemia was induced using Taira and Marsala’s method [16] by investigators blind to the group assignment. The left femoral artery was cannulated with a 2 Fr Fogarty catheter (Fogarty Arterial Embolectomy Catheter, Edwards Lifesciences, Irvine, CA, USA), a balloon tipped catheter. The catheter was inserted until it was place at descending thoracic aorta and the tip of the catheter was placed the left subclavian artery (about 11 cm from the insertion site). After cannulation, heparin 150 U was injected and the Fogarty catheter balloon was inflated with 0.05 mL of saline. Blood flow from the left carotid artery was simultaneously drained into the external reservoir to prevent proximal hypertension by withdrawing blood during the aortic occlusion. A successful aortic occlusion by the Foarty catheter was confirmed by an immediate and sustained decrease in the tail artery pressure.

After 10 min and 30 s of aortic occlusion, the Forgarty balloon was deflated, and the drained blood was reinfused to the left carotid artery. After finishing the procedure, all catheters were removed and incisions were closed. The rats were then allowed to recover from anesthesia and returned to their cages.

### Evaluation of neurobehavioral outcome

Neurological function was evaluated with hind limbs motor function after reperfusion by the observer who was blinded to the group assignments using motor deficit score (MDS) at 8 h, 1, 3, 5, and 7 days after reperfusion.

The MDS is measured as follows: 0 = normal; 1 = the animal walks normally, but legs are weak, and the animal cannot pull the legs if they are held by the examiner; 2 = the animal assumes normal body posture on a flat surface and is able to walk, but there is ataxia or spasticity; 3 = the animal is able to walk on its knuckles, or able to walk on the feet without proper stepping; 4 = the animal drags its legs, but there is movement at the knees; and 5 = the animal drags legs without significant movements in the lower limbs and either spasticity or flaccidity is present [17].

### Histopathology

After the last neurological examination, spinal cords were excised for histopathological examination. All rats were anesthetized again with mask-delivered isoflurane. The heart was exposed and the right auricle was cut with a 23-gauge needle inserted into the left ventricle. Heparinized saline was transcardially perfused passing through the needle and circulated around the body flowing out through the open right auricle. Spinal cord was separated and fixed in 10% buffered formalin for 24 h and L3–5 spinal cord segments were embedded in paraffin. Transverse sections were cut and stained with hematoxylin and eosin (H & E).

Neuronal injury was evaluated at X 200 magnification by the blinded investigator. Each slide was examined and the number of normal motor neurons in the anterior horn of spinal cord (anterior to a line drawn through the central canal perpendicular to the vertebral axis) was counted to assess the degree of ischemic neuronal injury. The spinal motor neuron with ischemic injury presents characteristic morphological feature of pronounced eosinophil cytoplasm, shrunken cell body, various degree of pericellular edema, and shrunken and darkly pyknotic nucleus. The anterior spinal cord of paraplegic animals was significantly destroyed with normal motor neurons reduced. The number of normal motor neurons was counted in 3 sections for each animal and averaged [18]. Double counting was avoided by careful examining the continuity of each cell.

### Statistical analysis

Statistical analysis was performed with the SPSS 21 (IBM Inc., Chicago, IL, USA). The normality of all the measured data was tested using Shapiro wilk test. Data were presented as mean (SD) or median (interquartile ranges [IQRs]). Repeated-measures ANOVA and post-hoc Bonferroni correction was used and, at each time point, MDS and the number of intact motor neuron were compared using Kruskal- Wallis tests followed by the Mann-Whitney U test. A Bonferroni correction was used to adjust type I error rate for multiple comparisons. The Bonferroni-adjusted *P* value was obtained by multiplying the unadjusted P value by the number of comparisons (i.e., 4), and was denoted by “corrected P.” A corrected *P*-value of less than 0.05 was considered to indicate statistical significance.

## Result

### Neurobehavioral outcome

All animals survived until the final neurological assessment at the 7th day after reperfusion. Hind limbs motor function was evaluated at 8 h, 1 d, 3 d, 5 d, and 7 d after reperfusion using MDS; The MDSs of a sham group (*n* = 10) was 0 (0) at each time point. The MDSs of the 4 experimental groups (the control group [group A] and the three treatment groups [group B, c and D] are shown in Table 1. When we compared the sham group and the 4 experimental groups, there was significant difference in MDSs at each time point (*P* < 0.001). Repeated measures ANOVA identified that there is a significant effect of time for MDS among the 4 experimental groups (*P* = 0.001). At post-reperfusion 8 h, no significant differences of MDS were observed among the 4 groups (3.5 [1.0] for group A, 3.0 [1.0] for group B, C and D; *P* = 0.846). At post-reperfusion day 1, Group D presented a significantly lower MDS compared with groups A, B, and C (3.0 [1.0] for group A, 3.5 [1.0] for group B, 4.0 [1.0] for group C and 3.0 [0] for group D; *P* = 0.006); this trend was sustained throughout the study period. There has been a significant change in MDS over time for group D (3.0 [1.0] at 8 h, 3.0 [0] at 1 day, 2.5 [1.0] at 3 and 5 day, 2.0 [1.0] at 7 day; *P* = 0.001), whereas no change was observed in MDS overtime for other groups during the study period.

**Table 1:** Motor Deficit Score (MDS) of the sham, control and treatment groups

Time after reperfusion	Group S (*n* = 10)	Group A (*n* = 10)	Group B (*n* = 10)	Group C (*n* = 10)	Group D (*n* = 10)	*P* value
8 h	0 (0) ¶	3.5 (1.0)	3.0 (1.0)	3.0 (1.0)	3.0 (1.0)	< 0.001
1 day	0 (0) ¶	3.0 (1.0)	3.5 (1.0)	4.0 (1.0)	3.0 (0)^*^	< 0.001
3 day	0 (0) ¶	4.0 (1.0)	4.0 (1.0)	4.0 (1.0)	2.5 (1.0)^* † ‡^	< 0.001
5 day	0 (0) ¶	4.0 (1.0)	4.0 (1.0)	4.0 (1.0)	2.5 (1.0)^* † ‡^	< 0.001
7 day	0 (0) ¶	3.5 (1.0)	4.0 (1.0)	4.0 (1.0)	2.0 (1.0)^* † ‡^	< 0.001

0 = normal; 1 = the animal walks normally, but legs are weak, and the animal cannot pull the legs if they are held by the examiner; 2 = the animal assumes normal body posture on a flat surface and is able to walk, but there is ataxia or spasticity; 3 = the animal is able to walk on its knuckles, or able to walk on the feet without proper stepping; 4 = the animal drags its legs, but there is movement at the knees; and 5 = the animal drags legs without significant movements in the lower limbs and either spasticity or flaccidity is present. Data are presented as median (IQR). Group S: sham group; Group A: control group; Group B: 0.5 mg/kg simvastatin group; Group C: 1 mg/ kg simvastatin group; Group D: 10 mg/kg simvastatin group

¶: P < 0.001 compared with Group A,B, C and D; *: *P* < 0.0125 compared with Group C; †: *P* < 0.0125 compared with Group B; ‡: P < 0.0125 compared with Group A;

### Histopathology

**Fig. 1:**
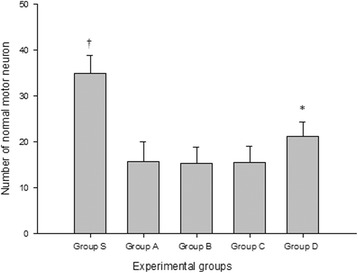
Normal motor neuron numbers in the anterior spinal cord. The number of normal motor neuron is significantly higher in group D than group B and C. Data are presented as mean (SD). Group S: sham group; Group A: control group; Group B: 0.5 mg/kg simvastatin group; Group C: 1 mg/ kg simvastatin group; Group D: 10 mg/kg simvastatin group. * *P* = 0.002 compared with group A, B and C. † *P* = 0.002 compared with group A, B, C and D

**Fig. 2:**
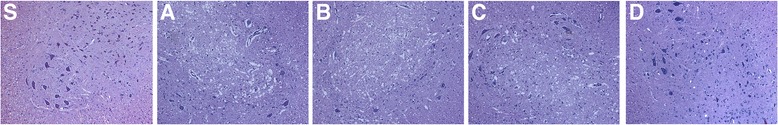
Representative microphotographs of the spinal cord from rats in each group. Group (**a**), group (**b**), and group (**c**) show similar features. Motor neurons suggest ischemic changes with shrunken nuclei and massive pericellular edema. Very few normal-looking motor neurons were observed. Grey matter shows spongy-like appearance due to marked vacuolization, and many infiltrating cells can be observed in remained gray matter. On the contrary, motor neurons of Group (**d**) show no massive vacuolization with minimal degree of pericellular edema. More intact motor neurons are observed. Group (**s**): sham group; Group (**a)**: control group; Group (**b**): 0.5 mg/kg simvastatin group; Group (**c**): 1 mg/ kg simvastatin group; Group (**d**): 10 mg/kg simvastatin group

The number of normal motor neuron of a sham group (*n* = 10) was 35 (3.8) and it was significantly higher than the other groups (*P* < 0.001 for each comparison). When we compared the control group and the three treatment groups, statistically significant difference was observed in the number of normal motor neuron among the 4 experimental groups. The number of viable motor neuron is significantly higher in group D compared with group A, B and C (group D 21.2 [3.2] vs. group A: 15.8 [4.2]; group B 15.4 [3.4]; and group C 15.5 [3.7]; *P* = 0.002, Fig. 1). Representative photos from each group are presented in Fig. 2.

## Discussion

In the present study, we reported the efficacy of simvastatin treatment administered after IR injury, for the first time in the literature. We showed that this simvastatin treatment after IR injury significantly improves the neurological outcome, as demonstrated by MDS and the number of normal motor neurons in a rat spinal cord ischemia model. Moreover, we determined that the most effective dosage of simvastatin treatment is 10 mg/kg simvastatin, which improved the neurologic outcome and increased the number of normal motor neurons in the anterior spinal cord after IR injury of the spinal cord.

Spinal cord injury leads to the loss of motor function in the hind limbs and a decrease in the number of normal motor neurons, which are closely correlated with the extent of spinal cord ischemia. Therefore, the neuroprotective effect of simvastatin post-treatment after IR injury was accompanied by low MDS and an increased number of normal motor neuron in spinal cord ischemia rats. Hwang et al., [14] investigated the pre-treatment effect of simvastatin after spinal cord ischemia, showing that 10 mg/kg simvastatin administered orally for 5 days before IR injury improved motor deficit index and preserved normal motor neurons. Gao et al., [19, 20] verified the molecular mechanism of the simvastatin neuroprotective effect and concluded that simvastatin inhibits neural cell apoptosis and preserved the motor neuron function by autophagy induction. Moreover, Saito et al., [15] investigated the treatment effect of 10 mg/kg simvastatin on hind limb motor dysfunction at 24 and 48 h after reperfusion in a rat spinal cord ischemia model. The current study investigated best dosage of simvastatin (0.5 mg/kg, 1 mg/kg and 10 mg/kg) as well as its long-term effect (7 days) after reperfusion in a rat spinal cord ischemia model.

In addition to its preventive neuroprotective effect, simvastatin treatment has previously been associated with improved functional neurologic outcome in a stroke model [21, 22]. The mechanism of statin’s neuroprotective effect after IR injury of the brain has not been fully elucidated to date, but seems to attribute to its anti-inflammatory and antioxidant effect [23]. These favorable CNS environments may create a potential microenvironment facilitating both regeneration of damaged neurons and remyelination of demyelinated axons after IR injury [21, 22]. Post-treatment of atorvastatin was also investigated in efforts to improve the recovery of motor functions after spinal cord injury and the attenuation of early inflammatory events by atorvastatin seemed to reduce secondary injury [24]. On the contrary, a few studies failed to observe the treatment efficacy of simvastatin after cervical or thoracic spinal cord ischemia [25, 26]. The possible explanation of these differences may be explained by the degree of severity of spinal cord ischemia, since these two studies used direct traumatic or contusion spinal cord ischemia model rather than an IR injury model.

The mechanism of IR injury includes damage induced by both ischemia and reperfusion. During the ischemic phase, inadequate oxygen supply and accumulation of toxic metabolites result in neuronal necrosis [27]. During the reperfusion stage, re-establishment of oxygen supply causes reactive oxygen species (ROS) formation and subsequent re-established blood supply facilitates neutrophils recruitment with immunologic cascade. During this process, various cytokines and growth factors aggravate tissue damage [27]. Therefore, pharmacological treatment of IR injury can prevent ischemic injury as well as the production of ROS with inflammatory cytokine. The mechanisms of neuroprotective effect of simvastatin after IR injury are not well recognized, but may be explained by anti-inflammatory effect, antioxidant effect, and vascular actions. First, simvastatin modulates endothelial nitric oxide synthase (eNOS), which improves cerebral blood flow in cells [28, 29]. Second, simvastatin has been known to reduce vascular inflammation, oxidative stress, and cytokine responses that occur during ischemia and reperfusion by reducing the induction of inducible nitric oxide synthase (iNOS), interleukin-1, and tumor necrosis factor-α in astrocytes and macrophages [30, 31].

Simvastatin was chosen for this study since simvastatin has a superior neuroprotective property compared with other statins due to a greater lipophilic property and more capability of crossing blood brain barrier [22]. The result of this study suggested that post-conditioning with 10 mg/kg simvastatin was neuroprotective after spinal cord IR injury, whereas 0.5 mg/kg and 1 mg/kg were not effective for the restoration of motor function. Simvastatin 0.5 mg/kg was based on the upper limit of 40 mg/day, considering its absorbed fraction (60–80%) and bioavailability (5%) with oral administration [32]. Simvastatin 1 mg/kg corresponds to 60–80 mg/day for the treatment of stroke patients [33]. Ischemic tolerance may vary depending on the species and brain region [34] and 10 mg/kg simvastatin was demonstrated to have preventive effect after IR injury of the spinal cord [14, 15], lung [8] and intestine [10]. Further studies are necessary to determine the optimal dosage for the application of human spinal cord IR injury.

This is the first in vivo experiment of simvastatin treatment after spinal cord IR injury with respect to the restoration of motor neuron and its function in a rat model. However, this has a few limitations to be considered. First, the result of this study suggested the neurologic outcome without the elucidation of the associated pathway or receptor. The mechanisms underlying the above-mentioned effects have not been fully elucidated and its anti-inflammatory, antioxidant effect and vascular actions seems to be responsible for this finding [28–31]. Second, larger doses could be necessary to reveal the optimal doses. The safe dose range was explored by administrating various doses to a healthy sham animal before the study. In a pilot study, 50 mg/kg was administered to the sham animal but some non-specific negative effects such as weight loss were found without histological change in spinal cord. Thus, simvastatin within the dosage range from the previous pilot study was tried in the current study. Further study is needed to determine the optimal dosage for the application of human spinal cord IR injury. Third, this therapeutic treatment of simvastatin was effective in rats when administered immediately after spinal cord ischemia. However, further studies are needed to better determine whether this treatment may be amenable for treating human patients.

## Conclusion

In summary, 10 mg/kg simvastatin treatment administered after spinal cord IR injury significantly improved the neurobehavioral outcome and preserved normal motor neuron in rats. The present findings suggest that simvastatin may be a promising therapeutic agent for the treatment of spinal cord IR injury.

## Abbreviations

CSF: Cerebrospinal fluid
eNOS: Endothelial nitric oxide synthase
H & E: Hematoxylin and eosin
HMG-CoA: Hydroxymethylglutaryl coenzyme-A
iNOS: Inducible nitric oxide synthase
IR: Ischemia-reperfusion injury
MDS: Motor deficit score
ROS: Reactive oxygen species
TAAR: Thoracoabdominal aortic aneurysm
TEVAR: thoracic endovascular aortic repair

## References

[1] Panthee N, Ono M (2015). Spinal cord injury following thoracic and thoracoabdominal aortic repairs. Asian cardiovasc thorac ann.

[2] Jonker FH, Trimarchi S, Verhagen HJ, Moll FL, Sumpio BE, Muhs BE (2010). Meta-analysis of open versus endovascular repair for ruptured descending thoracic aortic aneurysm. J Vasc Surg.

[3] Ullery BW, Wang GJ, Low D, Cheung AT (2011). Neurological complications of thoracic endovascular aortic repair. Semin Cardiothorac Vasc Anesth.

[4] Dias-Neto M, Reis PV, Rolim D, Ramos JF, Teixeira JF, Sampaio S (2017). Strategies to prevent TEVAR-related spinal cord ischemia. Vascular.

[5] Bicknell CD, Riga CV, Wolfe JH (2009). Prevention of paraplegia during thoracoabdominal aortic aneurysm repair. Eur J Vasc Endovasc Surg.

[6] Pereira JE, Costa LM, Cabrita AM, Couto PA, Filipe VM, Magalhaes LG, Fornaro M, Di Scipio F, Geuna S, Mauricio AC (2009). Methylprednisolone fails to improve functional and histological outcome following spinal cord injury in rats. Exp Neurol.

[7] Bao N, Ushikoshi H, Kobayashi H, Yasuda S, Kawamura I, Iwasa M, Yamaki T, Sumi S, Nagashima K, Aoyama T (2009). Simvastatin reduces myocardial infarct size via increased nitric oxide production in normocholesterolemic rabbits. J Cardiol.

[8] Pirat A, Zeyneloglu P, Aldemir D, Yucel M, Ozen O, Candan S, Arslan G (2006). Pretreatment with simvastatin reduces lung injury related to intestinal ischemia-reperfusion in rats. Anesth Analg.

[9] Lai IR, Chang KJ, Tsai HW, Chen CF (2008). Pharmacological preconditioning with simvastatin protects liver from ischemia-reperfusion injury by heme oxygenase-1 induction. Transplantation.

[10] Slijper N, Sukhotnik I, Chemodanov E, Bashenko Y, Shaoul R, Coran AG, Mogilner J (2010). Effect of simvastatin on intestinal recovery following gut ischemia-reperfusion injury in a rat. Pediatr Surg Int.

[11] Todorovic Z, Nesic Z, Stojanovic R, Basta-Jovanovic G, Radojevic-Skodric S, Velickovic R, Chatterjee PK, Thiemermann C, Prostran M (2008). Acute protective effects of simvastatin in the rat model of renal ischemia-reperfusion injury: it is never too late for the pretreatment. J Pharmacol Sci.

[12] Balduini W, De Angelis V, Mazzoni E, Cimino M (2001). Simvastatin protects against long-lasting behavioral and morphological consequences of neonatal hypoxic/ischemic brain injury. Stroke.

[13] Mahmood A, Goussev A, Kazmi H, Qu C, Lu D, Chopp M (2009). Long-term benefits after treatment of traumatic brain injury with simvastatin in rats. Neurosurgery.

[14] Hwang J, Han JI, Han S (2013). Effect of pretreatment with simvastatin on spinal cord ischemia-reperfusion injury in rats. J Cardiothorac Vasc Anesth.

[15] Saito T, Tsuchida M, Umehara S, Kohno T, Yamamoto H, Hayashi J (2011). Reduction of spinal cord ischemia/reperfusion injury with simvastatin in rats. Anesth Analg.

[16] Taira Y, Marsala M (1996). Effect of proximal arterial perfusion pressure on function, spinal cord blood flow, and histopathologic changes after increasing intervals of aortic occlusion in the rat. Stroke.

[17] Kanellopoulos GK, Kato H, Hsu CY, Kouchoukos NT (1997). Spinal cord ischemic injury. Development of a new model in the rat. Stroke.

[18] Umehara S, Goyagi T, Nishikawa T, Tobe Y, Masaki Y (2010). Esmolol and landiolol, selective beta1-adrenoreceptor antagonists, provide neuroprotection against spinal cord ischemia and reperfusion in rats. Anesth Analg.

[19] Gao K, Shen Z, Yuan Y, Han D, Song C, Guo Y, Mei X (2016). Simvastatin inhibits neural cell apoptosis and promotes locomotor recovery via activation of Wnt/beta-catenin signaling pathway after spinal cord injury. J Neurochem.

[20] Gao K, Wang G, Wang Y, Han D, Bi J, Yuan Y, Yao T, Wan Z, Li H, Mei X (2015). Neuroprotective effect of simvastatin via inducing the autophagy on spinal cord injury in the rat model. Biomed Res Int.

[21] Chen J, Zhang ZG, Li Y, Wang Y, Wang L, Jiang H, Zhang C, Lu M, Katakowski M, Feldkamp CS (2003). Statins induce angiogenesis, neurogenesis, and synaptogenesis after stroke. Ann Neurol.

[22] Karki K, Knight RA, Han Y, Yang D, Zhang J, Ledbetter KA, Chopp M, Seyfried DM (2009). Simvastatin and atorvastatin improve neurological outcome after experimental intracerebral hemorrhage. Stroke.

[23] Pannu R, Barbosa E, Singh AK, Singh I (2005). Attenuation of acute inflammatory response by atorvastatin after spinal cord injury in rats. J Neurosci Res.

[24] Pannu R, Christie DK, Barbosa E, Singh I, Singh AK (2007). Post-trauma Lipitor treatment prevents endothelial dysfunction, facilitates neuroprotection, and promotes locomotor recovery following spinal cord injury. J Neurochem.

[25] Lee JH, Tigchelaar S, Liu J, Stammers AM, Streijger F, Tetzlaff W, Kwon BK (2010). Lack of neuroprotective effects of simvastatin and minocycline in a model of cervical spinal cord injury. Exp Neurol.

[26] Mann CM, Lee JH, Hillyer J, Stammers AM, Tetzlaff W, Kwon BK (2010). Lack of robust neurologic benefits with simvastatin or atorvastatin treatment after acute thoracic spinal cord contusion injury. Exp Neurol.

[27] Zhu P, Li JX, Fujino M, Zhuang J, Li XK (2013). Development and treatments of inflammatory cells and cytokines in spinal cord ischemia-reperfusion injury. Mediat Inflamm.

[28] Endres M, Laufs U, Huang Z, Nakamura T, Huang P, Moskowitz MA, Liao JK (1998). Stroke protection by 3-hydroxy-3-methylglutaryl (HMG)-CoA reductase inhibitors mediated by endothelial nitric oxide synthase. Proc Natl Acad Sci U S A.

[29] Yamada M, Huang Z, Dalkara T, Endres M, Laufs U, Waeber C, Huang PL, Liao JK, Moskowitz MA (2000). Endothelial nitric oxide synthase-dependent cerebral blood flow augmentation by L-arginine after chronic statin treatment. J Cereb Blood Flow Metab.

[30] Pruefer D, Scalia R, Lefer AM (1999). Simvastatin inhibits leukocyte-endothelial cell interactions and protects against inflammatory processes in normocholesterolemic rats. Arterioscler Thromb Vasc Biol.

[31] Takemoto M, Liao JK (2001). Pleiotropic effects of 3-hydroxy-3-methylglutaryl coenzyme a reductase inhibitors. Arterioscler Thromb Vasc Biol.

[32] Shitara Y, Sugiyama Y (2006). Pharmacokinetic and pharmacodynamic alterations of 3-hydroxy-3-methylglutaryl coenzyme a (HMG-CoA) reductase inhibitors: drug-drug interactions and interindividual differences in transporter and metabolic enzyme functions. Pharmacol Ther.

[33] Flint AC, Conell C, Klingman JG, Rao VA, Chan SL, Kamel H, Cullen SP, Faigeles BS, Sidney S, Johnston SC (2016). Impact of increased early statin administration on ischemic stroke outcomes: a multicenter electronic medical record intervention. J Am Heart Assoc.

[34] Garcia JH, Liu KF, Ye ZR, Gutierrez JA (1997). Incomplete infarct and delayed neuronal death after transient middle cerebral artery occlusion in rats. Stroke.

